# Serum proteomic profiling and haptoglobin polymorphisms in patients with GVHD after allogeneic hematopoietic cell transplantation

**DOI:** 10.1186/1756-8722-2-17

**Published:** 2009-04-20

**Authors:** Joseph McGuirk, Gang Hao, Weijian Hou, Sunil Abhyankar, Casey Williams, Weisi Yan, Jianda Yuan, Xiuqin Guan, Robert Belt, Shaun Dejarnette, Jeffery Wieman, Ying Yan

**Affiliations:** 1 Blood and Marrow Transplantation Program, Saint Luke's Cancer Institute, Kansas City, Missouri, USA; 2 Department of Medicine, School of Medicine, University Missouri-Kansas City, Kansas City, Missouri, USA; 3 Department of Pharmacology, Weill Medical College of Cornell University, New York, New York, USA; 4 Laboratory of Cellular Immunobiology, Memorial Sloan-Kettering Cancer Center, New York, New York, USA; 5 Blood and Marrow Transplant Program, The University of Kansas Hospital Cancer Center, 2330 Shawnee Mission Parkway, Westwood, Kansas 66205, USA

## Abstract

We studied serum proteomic profiling in patients with graft versus host disease (GVHD) after allogeneic hematopoietic cell transplantation (allo-HCT) by two-dimensional gel electrophoresis (2-DE) and mass spectrometry analysis. The expression of a group of proteins, haptoglobin (Hp), alpha-1-antitrypsin, apolipoprotein A-IV, serum paraoxonase and Zn-alpha-glycoprotein were increased and the proteins, clusterin precursor, alpha-2-macroglobulin, serum amyloid protein precursor, sex hormone-binding globulin, serotransferrin and complement C4 were decreased in patients with extensive chronic GVHD (cGVHD). Serum haptoglobin (Hp) levels in patients with cGVHD were demonstrated to be statistically higher than in patients without cGVHD and normal controls (p < 0.01). We used immunoblotting and PCR in combination with 2-DE gel image analysis to determine Hp polymorphisms in 25 allo-HCT patients and 16 normal donors. The results demonstrate that patients with cGVHD had a higher incidence of HP 2-2 phenotype (43.8%), in comparison to the patients without cGVHD (0%) and normal donors (18.7%), suggesting the possibility that specific Hp polymorphism may play a role in the development of cGVHD after allo-HCT. In this study, quantitative serum Hp levels were shown to be related to cGVHD development. Further, the data suggest the possibility that specific Hp polymorphisms may be associated with cGVHD development and warrant further investigation.

## Introduction

Allogeneic hematopoietic cell transplantation (Allo-HCT) has been a potentially curative treatment approach for patients with hematological malignancies, lympho-hematopoietic failure, autoimmune diseases as well as genetic disorders. Despite its curative potential, the application of allo-HCT is limited by life-threatening complications, in particular, graft-versus-host disease (GVHD), a highly morbid toxic complication [1,2]. As a clinical syndrome related to the reaction of donor-derived immunocompetent cells against patient tissues, GVHD remains the most frequent transplant-related complication.

GVHD is classified as a clinicopathologic syndrome involving skin, liver, gastrointestinal tract, and/or other organs. Currently, there are no reliable laboratory tests that will confirm or refute its presence. Thus, GVHD is mostly a clinical diagnosis. Diagnosis of GVHD requires an interpretation of clinical and laboratory findings, recognizing that in some patients the differential diagnosis may be difficult to resolve [3]. To predict development and clinical prognosis of GVHD, several *in vitro *tests have been described. However, results have been difficult to reproduce and no assay has been widely adopted [3-7]. Studies of certain cytokine gene polymorphisms, including tumor necrosis factor alpha, interferon gamma, interleukin-1 (IL-1), IL-6 and IL-10, as well as polymorphisms of certain adhesive molecules such as CD31 and CD54 have been extensively conducted to explore their potential for GVHD risk prediction and the development of predictable genetic risk indexes. However, these efforts have not yet resulted in reliable models [3,8-13].

Over the past decade, the study of proteomics has rapidly evolved and developed. Proteomics studies can generate protein expression profiles which may predict clinical events, therapeutic response, or probe underlying mechanisms of disease. Proteome analysis is emerging as an important technology for understanding biological processes and discovery of novel biomarkers in diseases such as autoimmune disorders, cardiovascular diseases and cancers [14-17]. A recent study used an intact-protein-based quantitative analysis system for determining the plasma proteome profile of patients with acute GVHD after transplant. The proteins, including amyloid A, apolipoproteins A-I/A-IV and complement C3 were found to be quantitatively different between the pre- and post-GVHD samples [18]. In another report, several differentially excreted polypeptides were identified from patient urine samples by a capillary electrophoresis and mass spectrometry (CE-MS) based technique. The peptide profile displayed a pattern of early GVHD markers, allowing discrimination of GVHD from patients without the complication [19]. These reports hinted that GVHD can be monitored by changes in protein expression patterns detectable through proteomic methods.

Few investigations utilizing proteomic profiling in the study of patients with and without GVHD after allo-HCT have been reported to date. Several contributions in this regard have recently been reported to be confirmatory of a clinical diagnosis of acute GVHD (aGVHD) and to provide prognostic information. Paczesny et al have developed a panel consistent of 4 biomarkers which both confirm the diagnosis of aGVHD at onset of clinical symptoms and provide prognostic information independent of aGVHD severity [20]. Weissinger, et al have described an aGVHD-specific model consisting of 31 polypeptides and Hori et al have correlated a member of a large chemokine family, CCL8 to be closely correlated with aGVHD severity through proteomic analysis [21,22].

In this study, we performed serum proteomic profiling in a group of patients with and without cGVHD after allo-HCT by 2-dimensional electrophoreses (2-DE) and mass spectrometry based technology. Differential expression patterns of 11 serum proteins were demonstrated in patients before and after cGVHD development. Serum Hp precursors, one of the 11 differentially expressed serum proteins, were found to be significantly up-regulated during cGVHD development. We also investigated the relationship between serum Hp quantity as well as Hp polymorphisms and cGVHD development in this study. Serum Hp level as well as its polymorphisms were shown to be related to cGVHD development. Thus, Hp might serve as a worthy future target for monitoring cGVHD and understanding cGVHD mechanism.

## Methods

### Patients

Twenty-five patients who received allo-HCT at Saint Luke's Cancer Institute were studied. The 25 patients included 14 males and 11 females and the median age was 48 years (range 23–61 year old). Details of diagnostic indication for transplant are delineated in table 1. Sixteen patients developed cGVHD and 9 patients developed no cGVHD after allo-HCT. Samples were collected prior to transplant from each patient and at approximately 20 and 150 days, 6 months and 1 year or at the time of initial diagnosis of cGVHD (before initiation of steroid based therapy) in the BMT clinic and then periodically during follow up visits in patients with active cGVHD. Initial therapy of cGVHD included tacrolimus continuation or re-initiation and prednisone at 1 mg/kg daily. None of the 25 patients had a clinical diagnosis of transplant associated microangiopathy.

**Table 1:** Clinical characteristics of the allo-HSCT patients

	Sex/Age	Diagnosis	GVHD	Grade	Organ involvement	Conditioning Regimen	GVHD Prophylaxis	Infections	Outcome
1.*	F/40	AML	Chronic	Extensive	Skin eye oral	Cy/TBI/ATG	Tac/MTX	None	Alive, no active GVHD
2.*	M/28	CML	Chronic	Extensive	Liver	Bu/Cy	Tac/MTX	None	Alive, no active GVHD
3.	M/48	AML	Chronic	Extensive	Skin eyes oral gut	Bu/Cy	Tac/MTX	None	Alive, active GVHD
4.*	F/42	AML	Chronic	Extensive	Skin eye oral	Cy/TBI	Tac/MTX	None	Dead, AML relapse
5.*	F/42	IMF	Chronic	Extensive	Skin oral eye liver	Bu/Cy	Tac/MTX	None	Alive, active GVHD
6.	M/49	CLL	Chronic	Limited	Skin eye oral	Cy/TBI	Tac/MTX	None	Dead, GVHD
7.*	F/29	CML	Chronic	Extensive	Skin Gut	Cy/TBI	CSA/MTX	CMV	Dead, CMV pneumonia
8.*	F/58	AML	Chronic	Extensive	Skin Gut	Cy/TBI	CSA/MTX	None	Dead, unknown causes
9.*	F/45	AML	Chronic	Extensive	Skin	Cy/TBI	CSA/MTX	CMV	Dead, CMV/organ failure
10.*	M/38	CML	Chronic	Extensive	Eye oral gut	Bu/Cy	Tac/MTX	None	Alive, active GVHD
11.*	F/54	SAA	Chronic	Limited	Skin, oral	Cy/TBI/ATG	Tac/MTX	None	Alive, no active GVHD
12.*	M/54	NHL	Chronic	Extensive	Skin eye	Cy/TBI	Tac/MTX	None	Alive, active GVHD
13.	M/54	NHL	Chronic	Limited	Oral	BEAC	Tac/MTX	None	Alive, no active GVHD
14.*	M/50	NHL	Chronic	Extensive	Skin, lung	Cy/TBI/ATG	Tac/MTX	None	Dead, GVHD
15.	M/59	AML	Chronic	Limited	Skin Gut	Bu/Cy	Tac/MTX	CMV	Alive, active GVHD
16.	M/52	NHL	Chronic	Extensive	Skin Gut Oral	Cy/TBI/ATG	Tac/MTX	CMV	Alive, active GVHD
									
17.	M/29	CML	No GVHD	-	-	Cy/TBI	Tac/MTX	None	Alive, no active GVHD
18.	M/61	CLL	No GVHD	-	-	Bu/Flu/ATG	Tac/MTX	None	Alive, no active GVHD
19.*	F/40	AML	No GVHD	-	-	Cy/TBI/ATG	Tac/MTX	CMV, EBV	Alive, no active GVHD
20.	F/58	MDS	No GVHD	-	-	Cy/TBI	Tac/MTX	None	Dead, AML/MDS relapsed
21.*	M/38	AML	No GVHD	-	-	Cy/TBI/ATG	Tac/MTX	None	Dead, relapsed
22.	M/23	AML	No GVHD	-	-	Cy/TBI/ATG	Tac/MTX	None	Alive, no active GVHD
23.*	F/49	AML	No GVHD	-	-	Cy/TBI/ATG	Tac/MTX	None	Alive, no active GVHD
24.*	M/31	CML	No GVHD	-	-	Bu/Cy/ATG	Tac/MTX	None	Alive, no active GVHD
25.*	F/54	NHL	No GVHD	-	-	Cy/TBI/ATG	Tac/MTX	None	Dead, heart failure

AML: acute myelogenous leukemia; CML: chronic myelogenous leukemia; IMF: idiopathic myelofibrosis; CLL: chronic lymphocyte leukemia: SAA: severe aplastic anemia; NHL: Non-Hodgkin's Lymphoma; ALL: acute lymphoblastic leukemia; MM: multiple myeloma; MDS: myelodysplastic syndrome; * done of 2-DE gel assay.

Sixteen normal healthy donors, 10 males and 6 females, were included as controls in this study. Median age of the normal donors was 38 years (range 20–55).

### Serum processing

Peripheral blood samples were obtained, with informed consent, during routine diagnostic blood studies from patients before and after allo-HSCT in Saint Luke's Cancer Institute (SLCI). Serum samples were collected and aliquoted into 300 μl per tube from whole blood specimens, allowed to stand over night at 4°C without anti-coagulant and stored at -80°C in a freezer. Mononuclear cells (MNC) were isolated by Ficoll Hypaque density gradient separation and cyropreserved in liquid nitrogen. Serum albumin was removed by Swellgel Blue Albumin Removal kit (Pierce biotechnology, Rockford IL) following the instructions provided by the kit. 150 ul of serum was loaded for each single reaction. After preparing resin disc, binding sample and washing to release albumin-free sample, the albumin-free serum was collected for determination of protein concentration. The protein concentration was determined by the D_c _Protein Assay kit (Bio-Rad, Hercules CA) and following the instructions provided by the kit.

### 2-Dimensional protein gel electrophoresis

2-DE was performed as previously described with certain modifications [23]. Briefly, 500 μg of serum protein resuspended in rehydration buffer (8 M urea, 2% CHAPS, 0.5% immobilized pH gradient [IPG] buffer, 1% DTT, and trace of bromophenol blue) was loaded into an immobiline DryStrip (pI 4–7, 13 cm) (Amersham Biosciences) for rehydration over 18 hr. The first dimension isoelectric focusing was performed for 46,000 Vhr using a multiPhor II IEF System (Amersham) at 20°C. Then, the gels were equilibrated for 30 minutes in equilibration buffer I (50 mM Tris-HCL [pH 8.8], 6 M urea, 30% glycerol, 2% SDS, and 0.1% DTT) and buffer II (50 mM Tris-HCl [pH 8.8], 6 M urea, 30% glycerol, 2% SDS, and 0.25% iodoacetamide). The second dimension electrophoresis was conducted according to the Hoefer SE 600 system operating manual (Amersham). A gradient SDS-polyacrylamide gel (7%–12%) was used for the second dimension gel electrophoresis. The IPG strips were placed on the surface of the second dimension gel, and then the IPG strips were sealed with 0.5% agarose in SDS electrophoresis buffer (25 mM Tris base, 192 mM glycine, 0.1% SDS). The gels were run over 4 hrs at 110 V.

### Silver staining

Silver staining was performed according to a protocol published previously [23]. Briefly, gels were fixed with 50% methanol/10% acetic acid for 30 minutes and 5% methanol/1% acetic acid for 15 minutes respectively, and then the gels were washed by distilled water 3 times for 10 minutes each time. After washing, the gels were sensitized by incubation in sensitizing solution (0.02% sodium thiosulphate) for 90 seconds, and then rinsed with distilled water 3 times for 30 seconds each time. After rinsing, the gels were incubated in 0.2% silver nitrate for 30 minutes. The silver nitrate was then discarded and the gels were rinsed with distilled water 3 times for 1 minute each time and then developed with 0.02% formaldehyde and 0.0004% sodium thiosulphate in 6% sodium carbonate with shaking. The development was terminated with 6% acetic acid.

### Image analysis

The silver-stained 2-DE gels were scanned with LabScan software on an UMAX Powerlook III scanner (UMAX Tech Inc, California), and the images were digitalized and analyzed with a α-GelFox 2D 3.1 (Alpha Innotech) software.

### In-gel digestion and protein identification by LC-MS/MS

Protein spots were cut out from the silver-stained gels for in-gel digestion. Proteins were reduced and alkylated before digestion with trypsin (Promega, Madison, WI) overnight at 37°C. The peptides were extracted from the gel and concentrated in a vacuum centrifuge. 8 μL of concentrated peptide mixtures was injected to an Agilent LC-MSD ion trap mass spectrometer (Agilent Technologies, USA) for identification. Mass spectra were acquired in positive-ion mode with automated data-dependent MS/MS on the four most intense ions from precursor MS scans. The mass spectra were extracted and searched against the human database using Mascot software.

### Serum haptoglobin determination by Elisa Assay

Serum Hp determination was use AssayMax human Hp ELISA kit and followed the Elisa kit protocol provided by manufacturer (Assaypro St. Charles MO). Pooled human normal serum control (PNS), which contains serum derived from 20 normal donors, was purchased from George King Bio-Medical. INC. (Overland Park, KS).

### Haptoglobin genotype determination by PCR

Genomic DNA was extracted from peripheral blood MNC by the QIAamp DNA Kit as suggested by the supplier (Qiagen). Oligonucleotide primers A (5'-GAGGGGAGCTTGCCTTTCCATTG-3') and B (5'-GAGATTTTTGAGCCCTGGCTGGT-3') were used for amplification of a 1757-bp Hp-1 allele-specific sequence and a 3481-bp Hp-2 allele-specific sequence. Primers C (5'-CCTGCCTCGTATTAACTGCACCAT-3') and D (5'-CCGAGTGCTCCACATAGCC ATGT-3') were used to amplify a 349-bp Hp-2 allele-specific sequence [24]. The oligonucleotide primers were synthesized by IDT, Inc (Coralville IA). 20-μL reactions contained 2 U of *Taq *polymerase (Promega), 1–100 ng of DNA, and 200 μM each of dATP, dCTP, dGTP and dTTP (Promega); PCR buffer was used as suggested by the supplier (Promega) with no supplements added. After initial denaturation at 95°C for 2 min, the two-step thermocycling procedure consisted of denaturation at 95°C for 1 min and annealing and extension at 69°C for 2 min (in the presence of primers A and B or primers A, B, C, and D) or 1 min (in the presence of primers C and D only), repeated for 35 cycles, and followed by a final extension at 72°C for 7 min. The thermocycler used was Perkin Elmer 480 PCR system. For genotype assignments, the PCR products where primers A and B were used were separated in 1% agarose gels and products where primers C and D were used were separated in 8% polyacrylamide gels.

Restriction enzyme analysis was performed to verify the identity of Hp-1- and Hp-2-specific PCR products. The 1757-and 3481-bp products were digested with restriction enzyme *Mls*I, and the 349-bp product was digested with *Dra*I, as recommended by the supplier (MBI Fermentas). DNA fragments were separated by gel electrophoresis.

### Immunoblot

Immunoblot were performed as previously published with modifications [25]. Briefly, 1 μL of human serum in 20 μL of sample loading buffer [10 g/L sodium dodecyl sulfate (SDS), 100 mL/L glycerol, 25 mmol/L Tris (pH 6.8), 0.05 g/L bromphenol blue, and 50 mL/L β-mercaptoethanol] mixture were boiled at 95°C for 5 min, then the boiled samples were loaded on a 15% polyacrylamide gel. Standard Hp protein (Sigma Chemical Co.) was diluted to 1 g/L and treated in the same way as a control. Samples were electrophoresed in 25 mmol/L Tris base-192 mmol/L glycine-1 g/L SDS running buffer for 45 min at 150 V and then transferred to PVDF membranes (Bio Rad, California). The membranes were blocked in 5% Dry milk in Tris-buffered Tween [TBST; 10 mmol/L Tris-HCl (pH 8.0), 150 mmol/L NaCl, 0.5 mL/L Tween 20] for 1 h and then incubated at 4°C overnight with a 1:1000 dilution of polyclonal rabbit anti-human Hp antibody (Sigma). After washing the membranes three times in TBST, a second antibody, anti-rabbit IgG horse radish peroxidase conjugate (Santa Crus, California) was used at a dilution 1:2000 in TBST; the membranes were then incubated at room temperature for 1 h. The membranes were washed three times in TBST and finally developed with Luminal Reagent (Santa Crus).

### Statistical analysis

Statistical comparison of the serum Hp levels and 2-DE gel Hp protein spots VI among the different study groups was done using the Waller-Duncan K-ratio t test.

## Results

### Changes of protein expression patterns before and after cGVHD development

Thirty-six serum specimens derived from 16 patients and 9 samples from normal donors were subjected to 2-DE gel and silver staining assays. Out of the 36 patient-derived samples, 13 were collected before transplantation. Out of the 23 patient derived samples collected after allo-HCT, 9 samples were collected prior to the cGVHD occurring and 9 during cGVHD development, 5 were from the patients with no cGVHD development at all after receiving allo-HCT. The median number of protein spots in 2-DE gels was 709 (range 499–1012 spots) for the specimens from patients pre-transplantation; 637 (458–806 spots) in samples from prior to or no cGVHD development patients; 760 (508–1031 spots) in the serum from patients with active cGVHD and 735 (645–783 spots) in the serum of normal donors.

Paired 2-DE gel analyses were performed using 2-DE gel software between the serum collected prior to cGVHD and the samples of active cGVHD phase from the same individual patient. Protein spot patterns were significantly different between the gels of pre-cGVHD and active cGVHD phase in the same patient. The median protein spot numbers were determined as 753 (range 610–1012) and 726 (508–1031) for the specimens pre and post-cGVHD in the 7 patients with cGVHD, respectively. Median of matched spots between paired gels was 501 (405–725, median and range), however, in comparison to the gels pre-cGVHD, the medians for missing spots and newly appeared spots were 248 (190–391) and 276 (117–373), respectively in the gels of cGVHD.

**Figure 1:**
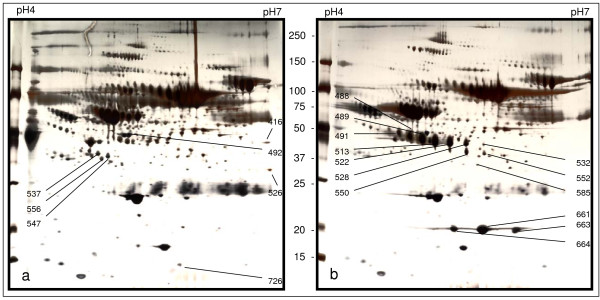
**A paired 2-DE gel images from a patient with cGVHD**. Gel a). represents the protein 2-DE gel profile of the serum from the patient before cGVHD development. Gel b). represents the protein 2-DE profile of the serum derived from the patient with cGVHD. The protein spots labeled with numbers were collected, digested and analyzed by Mass spectrometry.

**Figure 2:**
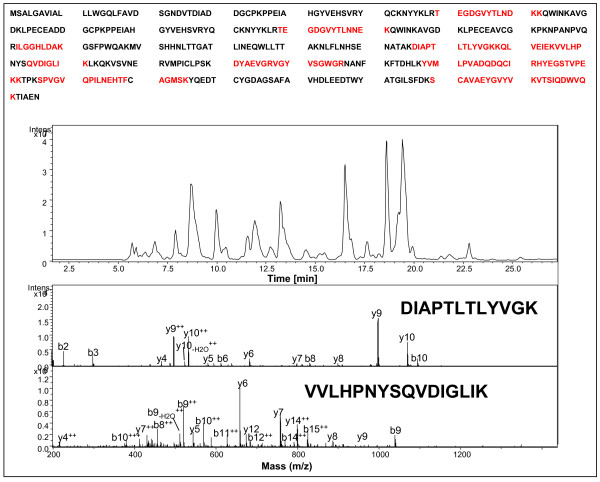
**Identification of haptoglobin by LC-MS/MS analysis and database searching**. Proteins were excised from the corresponding gel spots and subjected to in-gel digestion. The resulting peptides were extracted from the gel and analyzed by LC-MS/MS. MS/MS data were searched against the human database by Spectrum Mill software to obtain protein identification information. Upper panel: the sequence coverage of haptoglobin by LC-MS/MS analysis. Ten tryptic peptides (sequence underlined) were matched to human haptoglobin by database searching. Lower panel: MS/MS spectra of two of the ten peptides: DIAPTLTLYVGK and VVLHPNYSQVDIGLIK. The peptide sequences were established by extensive b and y ions matched to sequence.

A group of protein spots in the 2-DE gels was found significantly and consistently different between the serum collected prior to and during the cGVHD development from various patients. The protein spots, which were collected and analyzed from a paired representative 2-DE gels by LC-MS/MS are demonstrated in Figure 1. The sum of the spot areas multiple spot density as the volume index (VI) was used to determine the individual serum protein level in 2-DE gel semi-quantitatively. Five proteins, including Hp, apolipoprotein A-IV, α-1-antitrypsin, serum paraoxonase and Zn-α-glycoprotein were found quantitatively increased or newly appearing after cGVHD development. A group of 5 proteins were either down-regulated or absent in the 2-DE gel of patient with cGVHD, including sex hormone-binding globulin, clusterin precursor, serum amyloid A protein precursor, serotransferrin and complement C4 (Table 2). As a representative of protein spots analyzed, the mass spectrum identification of Hp by LC-MS/MS analysis is shown in Figure 2.

**Table 2:** Identity of proteins in 2-DE gel with increased or decreased intensity after onset of cGVHD

Accession No.	Spot No.	MW (KDa)	*p*l	Identified protein	(VI)/× 10^5^*	(VI) Fold change
P00738	489	46	4.8	haptoglobin-β	53.4	+4.2
	513	46	5.1	haptoglobin-β	47.1	+7.3
	522	45	5.2	haptoglobin-β	22.4	+6.5
	528	45	5.4	haptoglobin-β	6.5	+8.4
	552	40	5.6	haptoglobin-β	newly appeared	
	661	21	5.6	haptoglobin-α	22.8	+37.1
	663	21	5.9	haptoglobin-α	12.3	+32.0
	664	21	5.3	haptoglobin-α	7.4	+35.2
P06727	488	47	4.8	apolipoprotein A-IV	60.2	+25.5
	489	46	4.8	apolipoprotein A-IV	53.4	+4.2
	491	48	4.7	apolipoprotein A-IV	21.0	+15.0
P01009	489	46	4.8	α-1-antitrypsin	53.4	+4.2
	513	46	5.1	α-1-antitrypsin	47.1	+7.3
	528	45	5.4	α-1-antitrypsin	16.5	+8.4
P27169	488	47	4.8	serum paraoxonase	60.2	+25.5
	491	48	4.7	serum paraoxonase	21.0	+15.0
P25311	488	47	4.8	Zn-α-2-glycoprotein	50.2	+25.5
	491	48	4.7	Zn-α-2-glycoprotein	21.0	+15.5
P04278	492	48	5.1	sex hormone-binding globulin	1.2	-5.1
P10909	537	39	4.8	clusterin precursor	0.6	-4.5
	547	38	4.9	clusterin precursor	0.5	-4.9
	556	36	4.8	clusterin precursor	absence	
	726	13	5.6	clusterin precursor	0.1	-4.5
P02735	726	13	5.6	serum amyloid A protein precursor	0.1	-4.5
P02787	416	29	6.6	serotransferrin	absence	
P01023	556	36	4.8	α-2-microglobulin	0.2	-7.5
P01028	526	31	6.6	complement C4	absence	

Acession #: Number as listed in online protein database; VI: volume index

### Differential expression patterns of haptoglobin in cGVHD development

As a particular example, Hp expression patterns were demonstrated to be significantly different among the patients with and without cGVHD development in our 2-DE gel analysis. To quantitatively compare the differences in Hp spot volumes between the different study groups by 2-DE gel image analyses, we used VI to determine the serum Hp level semi-quantitatively. Since the complexity of Hp α chain polymorphisms and that the Hp β chain are identical in all haptoglobin phenotypes, we selected the VI of Hp precursor β as the representative of Hp volumes in each study groups. The VIs in the cGVHD group (n = 6), was demonstrated to be significantly higher than the VIs of the patients before transplantation (n = 12; p = 0.027); patients with no cGVHD after transplantation (n = 4; p = 0.045) and normal donor group (n = 9; p = 0.044), respectively (Table 3).

**Table 3:** Comparison of volume index (VI) of Hp spots in 2-DE between patients with and without cGVHD

Groups		Protein spot volume index (VI)/× 10^5 ^(Mean ± SD)	p- value
cGVHD Patients	(n = 6)	391.15 ± 305.38	
Before allo-HCT	(n = 12)	100.02 ± 108.89	0.03
After allo-HCT without GVHD	(n = 4)	146.49 ± 139.44	0.045
Normal donors	(n = 9)	136.71 ± 94.50	0.044

**Figure 3:**
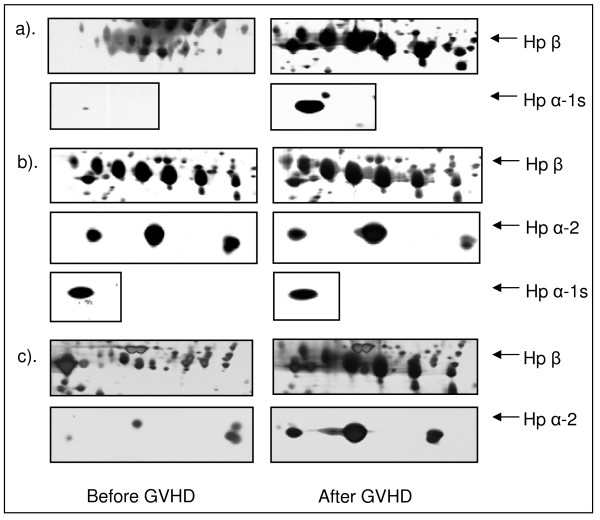
**Expression patterns of Hp in paired 2-DE gels in individual patients before and after GVHD**. The left panel lists the Hp 2-DE gel spots derived from patients before GVHD occurred and the right side panel represents the 2-DE gel spots after GVHD development.

The differential expression patterns of Hp in individual patients in paired 2-DE gels before and after cGVHD are demonstrated in Figure 3. Both volume and density of the Hp protein spots were shown to increase in the gels of cGVHD paired-set in all 3 patients irrespective of Hp phenotype differences. Figure 3a is from a cGVHD patient, who has an Hp β and an Hp α-1s chain, indicating an Hp 1-1 phenotype. The patient's Hp spot VI was 24.4 × 10^5 ^(Hp β) and 0.1 × 10^5 ^(Hp α-1s) before cGVHD and 295.1 × 10^5 ^(Hp β) and 29.4 × 10^5^(Hp α-1s) after cGVHD, respectively; 3b shows the 2-DE Hp pattern of a different cGVHD patient, who has Hp β, α-1s and α-2 chain which indicates a Hp 2-1 phenotype. The Hp spot VI was 91.0 × 10^5 ^(Hp β), 3.5 × 10^5 ^(α-1s) and 12.2 × 10^5 ^(α-2) before cGVHD and 140.9 × 10^5 ^(β), 4.8 × 10^5 ^(α-1s) and 18.7 × 10^5 ^(α-2) after cGVHD, respectively; Figure 3c represent the Hp expression patterns from another cGVHD patient. The patient expressed an Hp β and α-2 protein spots, suggesting an Hp 2-2 phenotype. The VI of 3c was 5.4 × 10^5 ^(Hp β) and 2.7 × 10^5 ^(α-2) before cGVHD and 274.9 × 10^5 ^(Hp β) and 67.7 × 10^5 ^(α-2) after cGVHD.

### Serum Hp levels in the patients before and after cGVHD development

**Figure 4:**
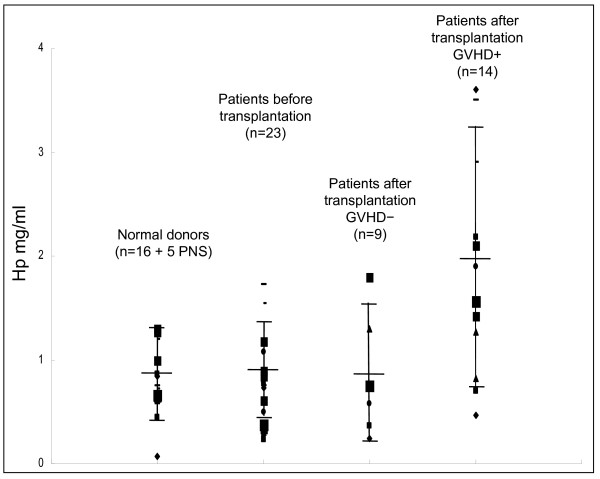
**Comparison of serum Hp level in patient with and without GVHD after allo-HCT by Elisa assay**. Serum Hp level in GVHD group is significantly higher than all the other 3 groups (p < 0.01)

We performed Elisa assays to confirm the prior finding and compared serum Hp levels in patients before and after cGVHD. Hp levels were examined in the serum from the same patient before and after cGVHD development, as well as normal donors. The mean of Hp concentration was 1.97 ± 0.99 mg/ml (mean ± SD) in the patients with cGVHD (n = 14); 0.83 ± 0.40 in the patients before transplantation (n = 23); 0.74 ± 0.51 in the patients with no cGVHD development after transplantation (n = 6) and 0.82 ± 0.31 mg/ml in the control group of normal donors (n = 16 and 5 PNS). Statistical analysis demonstrated that the serum Hp level in the cGVHD group was significantly higher than all the other 3 groups (p < 0.01). The differences of the serum Hp level were insignificant between the 3 cGVHD-negative groups (Figure 4).

### Determination of Hp polymorphisms in the patients

**Figure 5:**
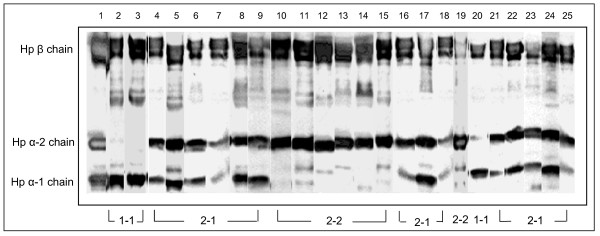
**Serum Hp phenotype determination of patients by immuoblot**. Polyclonal antibody against human Hp was used to binding the Hp on the blots. Lane 1 is the Hp protein standard containing Hp β, α-1 and α-2 chains. Lane 2–25 shows the serum Hp phenotype from patient 1 to patient 24 listed in Table 1. Patients who have Hp β and α-1 chains indicate a Hp 1-1 type; patients with Hp β and α-2 chains are Hp 2-2 type and patients with all the Hp β, α-1 and α-2 chains indicate a Hp 2-1 type.

**Figure 6:**
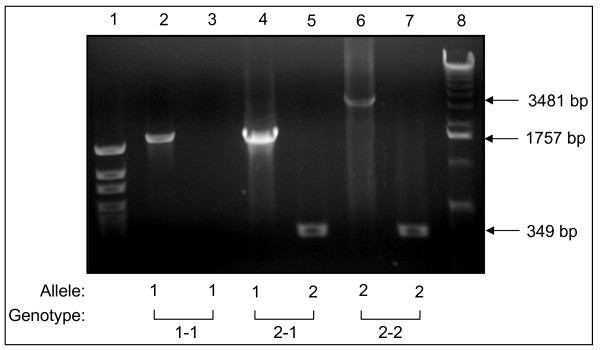
**Haptoglobin genotyping**. Hp genotyping based on combination of the results of two separate DNA amplification reactions involving primers A and B in the first reaction (lanes 2, 4, 6) and primers C and D in the second reaction (lanes 3, 5, 7). The reactions in lanes 2 and 3 contained DNA from the individual with genotype Hp 1-1; the reactions in lanes 4 and 5 contained DNA from the individual with genotype Hp 2-1; the reactions in lanes 6 and 7 contained DNA from the individual with genotype Hp 2-2. Lane 1, DNA size marker 100 – 1500 bp (Genscrip); lane 2, allele Hp 1; lane 3, no amplification product was obtained with the Hp 2-specific primer pair C/D because this sample was homozygous for allele Hp 1; lane 4, allele Hp 1; lane 5, allele Hp 2; lane 6, allele Hp 2; lane 7, allele Hp 2; lane 8, DNA size marker 1 kb plus (Gibco).

**Figure 7:**
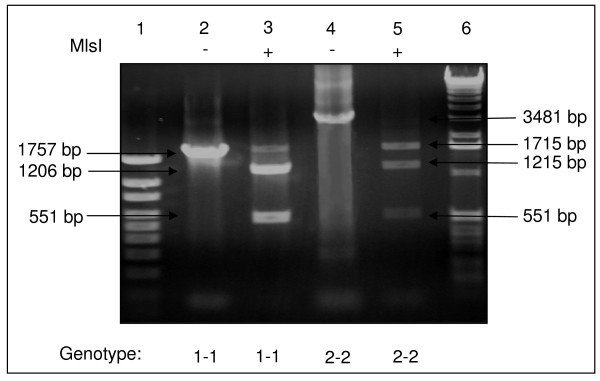
**Analysis of DNA amplification products representing genotypes with restriction enzymes *Mls*I**. Agarose gel showing experiments with *Mls*I. *Lane 1*, DNA size marker; *lane 2*, 1757-bp PCR product (Hp 1-specific), undigested; *lane 3*, 1757-bp product, digested with *Mls*I; *lane 4*, 3481-bp product (Hp 2-specific), undigested; *lane 5*, 3481-bp product, digested with *Mls*I; *lane 6*, DNA size marker.

**Figure 8:**
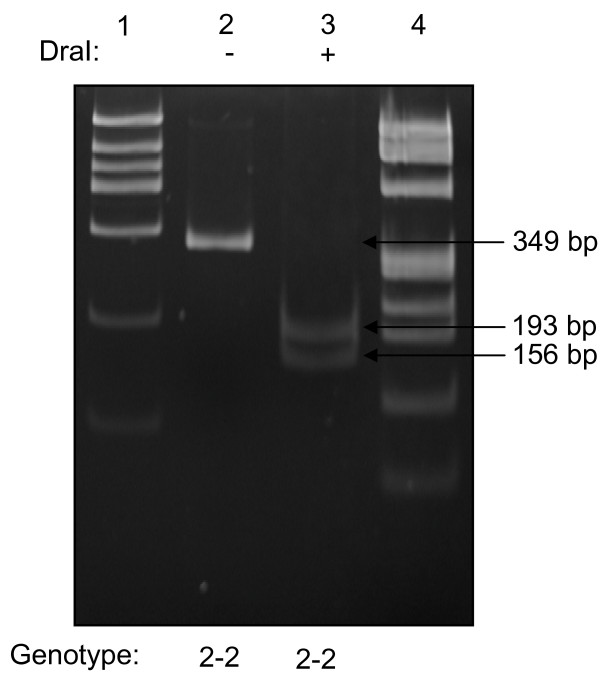
**Analysis of DNA amplification products representing genotypes Hp 2-2 with restriction enzymes *Dra*I**. polyacrylamide gel showing experiment with *Dra*I. *Lane 1*, DNA size marker; *lane 2*, 349-bp product (Hp 2-specific), undigested; *lane 3*, 349-bp product, digested with *Dra*I.

In humans, Hp is characterized by a molecular heterogeneity with three main genotypes/phenotypes: Hp 1-1, Hp 2-1, and Hp 2-2. These different proteins have distinctive efficiencies and it has been suggested that the polymorphism may have important biological consequences in several diseases [26]. To probe the possible relationship between cGVHD and Hp polymorphism, we used immunoblot and PCR in combination with 2-DE gel image analysis to determine Hp polymorphisms in the 24 allo-HCT patients of this study group as well as 12 normal donors. Serum Hp phenotype was determined by immunoblot using polyclonal antibody, which recognize Hp β, α-1 and α-2 chains and the Hp phenotypes in the patients are shown in Figure 5. To verify the Hp phenotype in these patients, we examined Hp genotypes of the patients by PCR assay (figure 6), and the Hp PCR products were further confirmed by restriction enzyme analysis (figure 7 and 8). All the DNA typing of the samples derived from 25 patients were matched with their phenotypes determined by immunoblot.

Out of 16 patients with cGVHD, 2 (12.5%, 1 limited and 1 extensive) had an Hp 1-1 type; 7 (43.8%, 4 extensive and 3 limited) were 2-1 and 7 (43.8%, 6 extensive and 1 limited) were Hp 2-2 type (Table 4). In comparison with normal donors, patients with cGVHD had a higher incidence of Hp 2-2 phenotype (43.8%) and a lower incidence of Hp 2-1 (43.8%) type than the normal donors (18.7%, p < 0.01) and (75.0%, p < 0.05), respectively. In addition, out of 9 patients in whom no cGVHD occurred, 8 (88.9%, p < 0.01) were Hp 2-1 type and 1 (11.1%) was 1-1 type, but no 2-2 type was detected (Table 4), suggesting that the patients with Hp 2-2 phenotype might have more genetic susceptibility or tendency for cGVHD development.

**Table 4:** Serum Hp polymorphism in the patients and normal controls

		**Hp phenotype**		
**Patients**		**1-1**	**2-1**	**2-2**
Chronic GVHD	n = 16	2(12.5%)	7(43.8%)	7(43.8%)
no GVHD	n = 9	1(11.1%)	8(88.9%)	0
Normal Donor	n = 16	1(6.3%)	12(75.0%)	3(18.7%)

## Discussion

Proteome analysis is now emerging as an important technology for deciphering biological processes and is aiding in the discovery of biomarkers for diseases from tissues and body fluids. In this study, we examined serum proteomic profiles in a group of patients with cGVHD after allo-HCT by two-dimensional gel electrophoresis (2-DE) and mass spectrometry based technology. A panel of proteins, Hp alpha-1-antitrypsin, apolipoprotein A-IV, serum paraoxonase and Zn-alpha-glycoprotein were demonstrated to be up-regulated and clusterin precursor, alpha-2-macroglobulin, serum amyloid protein precursor, sex hormone-binding globulin, serotransferrin and complement 4 were found down-regulated in the patients with cGVHD.

Medical literature in the area of proteomic profiling in GVHD is scarce [18,19,27]. One study has used an intact-protein-based quantitative analysis combined with protein tagging and immunodepletion of abundant proteins to quantitatively profile the plasma proteome in the patients with acute GVHD after transplant [18]. In this study, plasma samples were subjected to immunodepletion chromatography to remove six of the most-abundant plasma proteins (albumin, transferrin, IgG, IgA, Hp and α-1-antitrypsin) to increase the sensitivity of serum low abundant protein detection. However, it is not clear whether or not serum high abundant proteins such as Hp, transferrin and immunoglobin, est., are involved in the pathophysiology of cGVHD. In our study, high abundant proteins including Hp, alpha-1-antitrypsin and transferrin exhibited quantitative differences between the pre- and post-GVHD samples, which suggest that those proteins might be importantly involved in the pathophysiologic processes of cGVHD. Therefore, the potential role of these high abundant proteins in the development and propagation of cGVHD should be fully assessed before being methodically eliminated in proteomic profiling studies. Increased serum Zn-alpha-glycoprotein and decreased complement C4 in patients with cGVHD in our study were in agreement with this report [18]. In contrast, serum amyloid protein and alpha-2-macroglobulin, which were increased in their study, were down-regulated in our study [18]. One possible explanation might be that the immunodepletion process affected their results.

In our study, Hp was identified as one of the increased proteins after cGVHD onset. Both the results of Hp volume index in 2-DE gel image analysis and serum Elisa assay demonstrated a significant increase of Hp in patients with cGVHD. Hp is an acute-phase response serum protein that has been known to play an important inhibitory role in inflammation and the Hp plasma concentration may increase in response to a variety of stimuli, such as: infection, neoplasia, and other inflammatory and immune reactions [28-30]. In this study, we report for the first time that an increase of serum Hp concentration is observed in patients with cGVHD after allo-HCT. The quantitative changes of serum Hp, as well as the well known acute-phase reactants found in this study, such as apolipoproteins A-IV, complement C4 and serum amyloid A thus might reflect changes in these proteins as manifestation of their roles in the pathophysiologic development and propagation of cGVHD or, alternatively, simply a nonspecific manifestation of an inflammatory state. Other investigators have described results that differ from the data reported here in terms of some acute phase reactants such as apolipoproteins in the setting of GVHD [31]. However, these data were derived from patients undergoing cord blood transplantation in contrast to our data set which is derived from patients receiving only adult derived hematopoietic stem cell transplantation. Additionally, the samples were collected within the first 100 days of transplant, before cGVHD could have developed in the report of Harvey, et al. Finally, the subtype of apolipoprotein measured differed from our study. Hp levels may increase in response to various stimuli, a further well designed study with more cases included would be necessary for ruling out the Hp changes secondary to transplantation-related infection, lung injury, and other possible complications post-transplant. Additionally, haptoglobin levels may be significantly changed in patients experiencing GVHD associated microangiopathy [32]. Although, no patient in our data set met criteria for transplant associated microangiopathy, this represents a well described GVHD associated clinical syndrome and will need to be closely evaluated in future studies concerning the association of haptoglobin and cGVHD.

Hp is characterized by molecular heterogeneity with three major phenotypes: Hp 1-1, Hp 2-2, and the heterozygous Hp 2-1 [33-35]. Hp is synthesized as a single polypeptide chain and is proteolytically cleaved to a short α-chain and a long β-chain that remains connected through a disulfide bond. Although Hp is found in serum of all mammals, this polymorphism exists only in humans [36,37]. These Hp phenotypes have different biologic activities, which include a stronger anti-oxidation, hemoglobin banding and anti-·OH production activities derived from Hp 1-1 and otherwise a stronger activity of macrophage activation for Hp 2-2 [23,27,36,37]. The functional differences between Hp phenotypes may play a role in determining the severity and extent of myocardial damage in the setting of myocardial infarction; Hp 2-2 is considered an independent predictor of myocardial infarction [38,39]. The Hp 1-1 phenotype was reported to be protective in the setting of two critical vascular complications of diabetes mellitus: diabetic nephropathy and restenosis after percutaneous transluminal coronary angioplasty [40]. In addition, Hp 2-2 was reported to be overrepresented in autoimmune diseases, such as rheumatoid arthritis and systemic lupus erythematosus [41,42]. In our small case study, 43.8% of patients with cGVHD had an Hp 2-2 phenotype, higher than the normal donor group (Hp 2-2 18.7%). In addition, no Hp 2-2 type was found in the 9 patients with no clinical cGVHD presentation after allo-HCT (Table 4). Based on our preliminary results and the characteristics of the lower anti-oxidation activity and higher potential of APC cell activation by Hp 2-2, we may suspect that the patients with Hp 2-2 phenotype might have more genetic susceptibility or tendency for cGVHD development than the patients with Hp 1-1 or 2-1 type. To further confirm this hypothesis, a prospective analysis of correlations of Hp phenotype and the subsequent development of cGVHD is now being conducted.

In conclusion, we found that an increase in Hp expression is associated with cGVHD development. Further, the Hp 2-2 phenotype is present in patients who develop cGVHD more commonly than in those who do not develop this immunologic complication after allo-HCT. These findings might establish Hp as a valuable protein candidate for early cGVHD prediction and diagnosis. Several recently reported studies have utilized proteomic profiling in the development of predictive models of aGVHD [20-22]. Optimally, further studies utilizing proteomic profiling in patients with cGVHD will eventually lead to predictive models as well. Finally, further studies involving many more patients regarding the possible effects of Hp on T cell function during cGVHD are highly desirable.

## Competing interests

The authors declare that they have no competing interests.

## Authors' contributions

JM carried out the clinical research and participated in the design of the study. SA, CW and RB carried out the clinical research and participated in clinical patient care. GH and WY participated in In-gel digestion and protein identification by LC-MS/MS. WH and JY carried out 2-Dimensional protein gel electrophoresis, silver staining, Haptoglobin genotype determination by PCR and immnoblot. XG performed Serum haptoglobin determination by Elisa Assay. SD carried out clinical date collection and coordinated patient specimen collection. JW and YY participated and conceived in the design of the study and coordination. All authors read and approved the final manuscript.
